# Projection of American dustiness in the late 21^st^ century due to climate change

**DOI:** 10.1038/s41598-017-05431-9

**Published:** 2017-07-17

**Authors:** Bing Pu, Paul Ginoux

**Affiliations:** 1 Atmospheric and Oceanic Sciences Program, Princeton University, Princeton, New Jersey 08544 USA; 2 NOAA Geophysical Fluid Dynamics Laboratory, Princeton, New Jersey 08540 USA

## Abstract

Climate models project rising drought risks over the southwestern and central U.S. in the twenty-first century due to increasing greenhouse gases. The projected drier regions largely overlay the major dust sources in the United States. However, whether dust activity in U.S. will increase in the future is not clear, due to the large uncertainty in dust modeling. This study found that changes of dust activity in the U.S. in the recent decade are largely associated with the variations of precipitation, soil bareness, and surface winds speed. Using multi-model output under the Representative Concentration Pathways 8.5 scenario, we project that climate change will increase dust activity in the southern Great Plains from spring to fall in the late half of the twenty-first century – largely due to reduced precipitation, enhanced land surface bareness, and increased surface wind speed. Over the northern Great Plains, less dusty days are expected in spring due to increased precipitation and reduced bareness. Given the large negative economic and societal consequences of severe dust storms, this study complements the multi-model projection on future dust variations and may help improve risk management and resource planning.

## Introduction

Climate models project increases of drought conditions in the southwest and central Great Plains in the late twenty-first century^1, 2^, co-located with the major dust sources in the United States (U.S.). If these models are correct, will the level of “unprecedented” aridity in the next half of this century^2^ produce more dust storms in the future? Given the severe health and social impact of the dust storms (e.g., refs 3, 4) several model studies have attempted to estimate future projections of dust on a global scale, with uncertain results (e.g., refs 5, 6). Unfortunately, none of these studies provide specific information for the U.S. One reason is the difficulty for models to properly reproduce observed dust variability over North America^7^. Solutions have been proposed, for example, to modulate dust source strength to fit the data^7^. But such method cannot project future dust change. Here we perform a multiple linear regression analysis of key controlling factors of dust activity using satellite data. We then use projected changes of these controlling factors from the Coupled Model Intercomparison Project Phase 5 (CMIP5^8^) models to project future dust activity due to climate changes in the late half of the twenty-first century under the Representative Concentration Pathways 8.5 (RCP8.5) scenario^9^. The RCP 8.5 scenario represents the upper limit of the projected CO_2_ changes in the twenty-first century and, presumably, would be the worst-case scenario of severe droughts and resultant dust activities.

## Results

### Variability of dust event frequency in the recent decade

**Figure 1:**
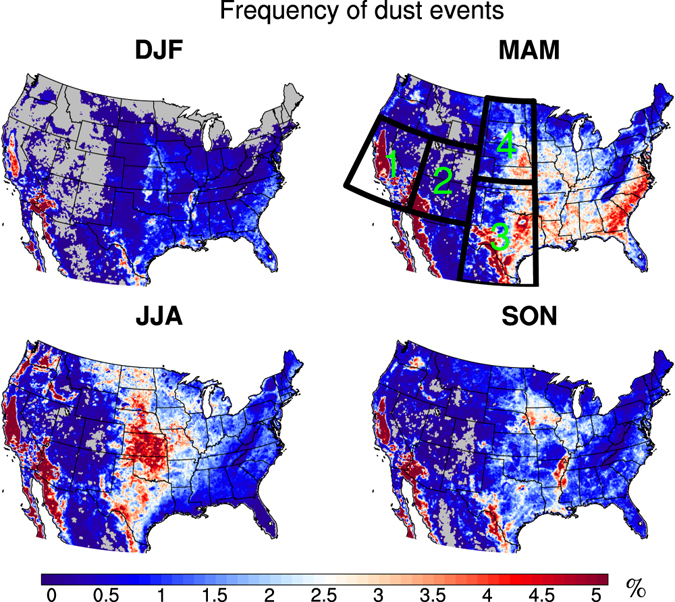
Climatology (2003–2015) of the frequency of dust events. Black boxes denote the averaging areas: Box 1 for the southwestern U.S. (WST1; 32°–42°N, 114°–124°W), Boxes 1 and 2 for the western U.S. (WST; 32°–42°N, 105°–124°W), Box 3 for the southern Great Plains (SGP; 25°–38°N, 95°–105°W), and Box 4 for the northern Great Plains (NGP; 38°–49°N, 95°–105°W). Missing values are shaded in grey. Maps were generated using the National Center for Atmospheric Research (NCAR) Command Language (NCL; https://www.ncl.ucar.edu/), version 6.2.1.

Figure 1 shows the climatology (2003–2015) of the frequency of dust events over the contiguous U.S., derived from daily dust optical depth (DOD; see Methods) from the Moderate Resolution Imaging Spectroradiometer (MODIS) Aqua platform, in each season. The high frequency over California, Arizona and northern Mexico largely matches the dust sources (such as the Mojave, Sonoran, and Chihuahuan deserts) in these regions. The hot spot over the Great Plains in spring and summer is associated with wind erosion in farmland and a few dry lakes, whereas in the southern Great Plains and the Gulf coast transported dust from Africa and northern Mexico can also contribute to the high frequency (Fig. 1). Note that although the frequency over the eastern coast in spring is comparable to that over the Great Plains, the magnitude of those events is much lower, as the background DOD is lower in the eastern U.S. (Supplementary Fig. S2). Over the southwestern U.S. (Box 1 in Fig. 1) and the northern Great Plains (Box 4), strong dust events usually occur in spring, while in the southern Great Plains (Box 3), summer is the dustiest season (Fig. 1 and Supplementary Fig. S3).

**Figure 2:**
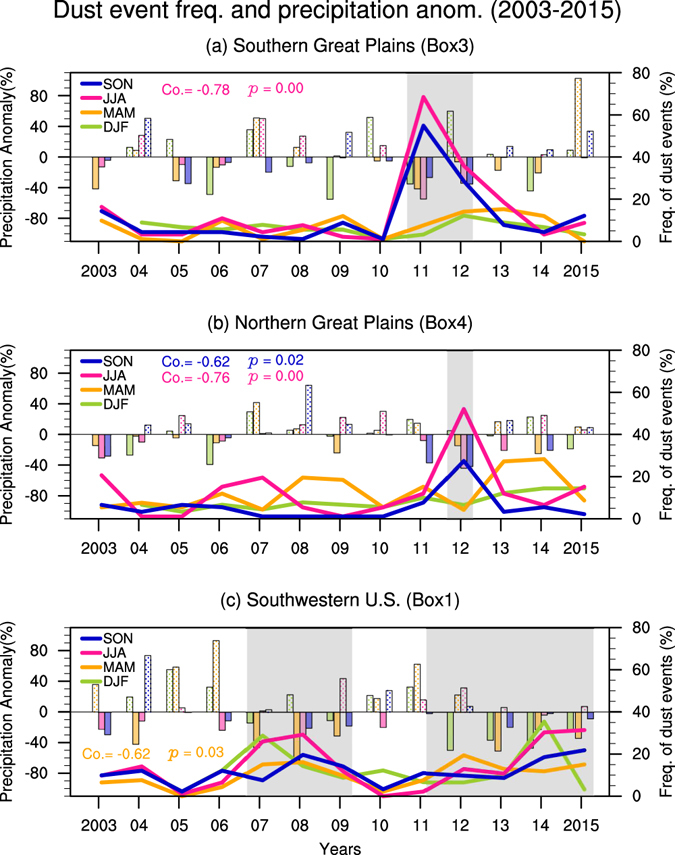
Dust event frequency and precipitation anomaly. Time series of the dust event frequency (%; lines) and precipitation anomaly (in percentage with reference to the 2003–2015 climatology; bars) in the southern Great Plains (SGP; Box 3 in Fig. 1), northern Great Plains (NGP; Box 4), and the southwestern U.S. (Box 1) for each season (light green for winter, orange for spring, magenta for summer, and navy blue for fall). Recent drought years are shaded in grey. Correlation coefficients between the precipitation and dust event frequency above the 95% confidence level (t-test) are listed (color denoting seasons).

Will drought intensify dust activities in the U.S.? We first examine how precipitation variations influence dust activities. Precipitation can reduce dust activities by scavenging airborne dust and increasing soil moisture in the dust source region (e.g., ref. 10), prohibiting the uplifting of dust particles by winds, and therefore precipitation deficits usually favor dust activity (e.g., refs 11, 12). Figure 2 shows the dust event frequency from 2003 (2004 winter) to 2015 along with precipitation anomalies (with reference to the climatology) in each season. In the recent decade, several severe droughts have been reported, such as the 2011 drought in the southern Great Plains, 2012 drought centered in the central Great Plains, and the California droughts from late 2011 to 2015 (also extending to 2016) and a slightly weaker dry period from 2007 to 2009 (e.g., refs 13–16). The maxima of dust event frequency match these dry episodes quite well (grey shading in Fig. 2). We also found there are positive linear trends of dust event frequency in the northern Great Plains in winter (p < 0.002) and spring (p = 0.09), and over the southwestern U.S. in spring (p = 0.07), which is to some extent consistent with the recent positive trends of dust emissions found in the western and southwestern U.S.^17, 18^, although different time periods were examined in these studies. Due to the short time coverage of the data, it is hard to determine whether the positive trends are associated with any long-term trend or just a part of decadal variations.

In addition to precipitation, surface wind and surface bareness can also contribute to the variability of dust emission and transport (e.g., refs 18–21). To examine the connections between dust event frequency and these potentially influential factors, a multiple-linear regression is applied to the seasonal averaged precipitation, surface wind speed, and bareness to examine their contribution to seasonal dust event frequency.

While *in-situ* and laboratory measurements indicate that dust emission is related to the third power of surface wind speed, studies also suggest that monthly dust emissions and transport have good linear relationships with monthly wind speed^19^. In this study we do not separate the contribution from local emissions or remote transportation to local dust load, although Asian contributions have been observed in spring in the western U.S.^22, 23^ and North African contributions in summer in the Southeast^24, 25^. Seasonal mean wind speed is used. We also tested using wind speed frequency calculated from daily surface wind speeds, and the results are similar. The presence of non-erodible elements, such as vegetation, is a major factor in reducing soil erosion by wind^26^. Here, surface bareness is derived from leaf area index (LAI) and describes the vegetation coverage. Another controlling factor of dust emission is soil moisture^27^, but is estimated over different depths across models. An alternative is precipitation^28^. While the scavenging process happens on a very short time scale, e.g., hours to a few days, the influence of precipitation on drought condition lasts much longer, e.g., weeks to months (refs 29, 30), so we chose to use seasonal precipitation instead of a shorter time scale. We also examined two cases using daily perception data (see discussion below) and those results are consistent with seasonal regressions.

**Figure 3:**
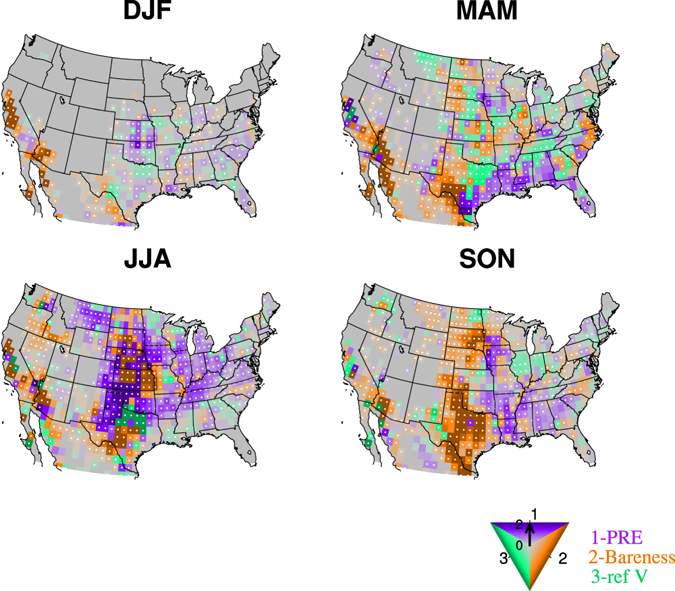
Dominant contributors to the variations of dust event frequency. Regression coefficients are obtained by regressing the dust event frequency in each season onto the standardized precipitation (purple), bareness (orange), and surface wind speed (green) from 2004 to 2015. The color of the shading denotes the largest coefficient in absolute value among the three while the saturation of the color shows the magnitude of the coefficient (from 0 to 2). Regression coefficients significant at the 90% confidence level (Bootstrap test) are dotted. Missing values are shaded in grey. Maps were generated using the NCAR Command Language (NCL; https://www.ncl.ucar.edu/), version 6.2.1.

Figure 3 shows which one of the three factors contributes the most to dust event frequency over the United States for each season. Over the Great Plains, the dominant factors affecting dust event frequency in winter are precipitation and surface wind speed. In spring, surface wind becomes more important, along with bareness. The former is related to the development of the Great Plains low-level jet (e.g., refs 31–35) in April and May, and the latter is associated with the start of the growing season in the Great Plains (Supplementary Fig. S4). Spring precipitation is also important over the southern Great Plains. In summer, precipitation and bareness are two dominant factors influencing the dust event frequency in the Great Plains, while in fall bareness is the most influential factor.

Only a narrow band over the southwest shows a relatively strong linear relationship between dust event frequency and the three factors. Bareness plays the most important role over central California in winter and over southern California from spring to fall. Other factors such as surface wind and precipitation seem to have weaker connections with dust event frequency. One explanation is that precipitation is relatively low during the dusty seasons in spring and summer (Supplementary Fig. S4), and thus vegetation growth becomes the most influential factor limiting dust events. On the other hand, variations of bareness also reflect the influence of precipitation in previous seasons. For instance, summer bareness is significantly negatively correlated with spring precipitation in this region. Some missing values also may limit our regression analysis in this region, such as in northeast California due to the missing data of LAI.

**Figure 4:**
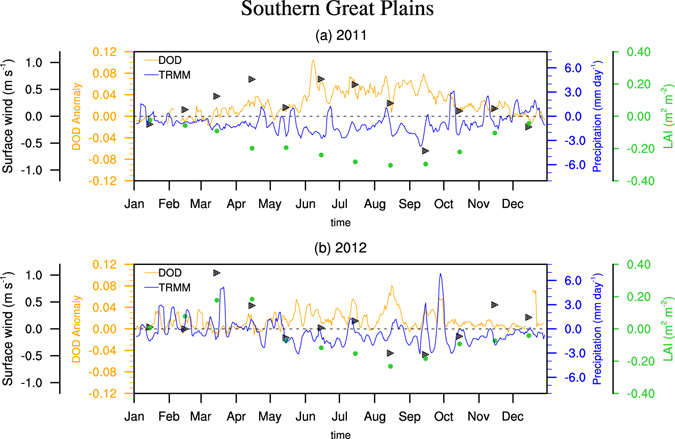
Case studies of two dry years in the southern Great Plains. Daily MODIS Aqua DOD and TRMM precipitation anomalies (with reference to the mean of 2003–2015) are plotted in orange and blue lines, respectively, while monthly anomalies of NARR surface wind and the bareness calculated from AVHRR LAI are plotted in black triangles and green dots, respectively. Five-day running mean is applied to daily precipitation and DOD time series.

How well the regression analysis resembles the physical connection between dust activity and the three influential factors is examined for recent severe drought years in the southern Great Plains (Fig. 4). During the 2011 drought, the decrease of precipitation is accompanied by an increase of DOD from spring to fall. The increase of bareness generally follows the precipitation deficit and is consistent with the increase of DOD. Surface wind also increases in spring, summer and late fall, facilitating the emission and transport of dust particles. Precipitation deficiency over the southern Great Plains is weaker in 2012, and so are the variations of DOD and bareness, while the contribution from the surface wind is mainly in early summer and late fall.

The regression model can largely capture the interannual variation of dust event frequency during 2004–2015 (Supplementary Fig. S5). The correlation between the calculated dust event frequency and that of Aqua ranges from 0.70 in fall to 0.91 in summer in the western U.S., and from 0.84 in winter to 0.94 in summer in the Great Plains, indicating that the three controlling factors (precipitation, bareness, and surface wind speed) can statistically explain 49–83% of the variances of dust event frequency in the western U.S. and 71–88% in the Great Plains during the recent decade.

It is possible that the strength of the relationships among dust, precipitation, surface wind, and bareness established in the recent decade will vary over a longer time period. For instance, during very dry episodes, the occurrence of dust events may be more sensitive to wind speed but less so to lower-than-normal precipitation as the surface is already quite dry. However, an overall negative connection between dust event frequency and seasonal mean precipitation and positive connections with surface wind speed and bareness should still largely hold.

Another important factor associated with dust emission but not considered here is the change of land use/land cover. For instance, the devastating Dust Bowl in the 1930s was caused by a combination of persistent drought, land use/land cover change, i.e., grasslands to non-irrigated croplands decades before the Dust Bowl and losses of vegetation cover during the Dust Bowl, improper farming methods (such as “dust mulch”) that increased the erodibility of top soil, and economic factors^36^. LAI used in the regression model shows the overall vegetation density but provides no specific information regarding to the type of land cover or the use of land. The short time period examined here also prohibits any further analysis on the change of land use/land cover on dust emission, which could take a much longer time (e.g., several decades). Therefore, the following analysis focuses on changes of dust activity associated with climate change and variability.

### Projection of dust event frequency change in the late half of the twenty-first century

**Figure 5:**
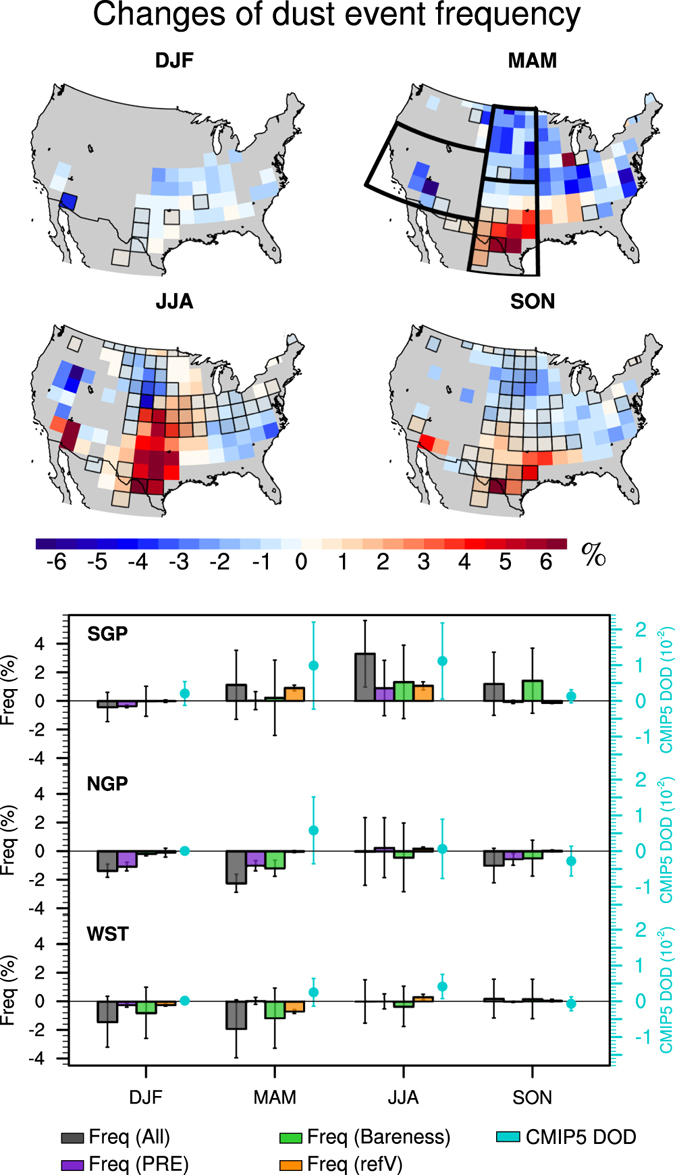
Changes of dust event frequency in the late half of the twenty-first century (with reference to the historical run, 1861–2005). Shaded grids in the upper panel denote area where the agreement among the models is lower than 62.5% (see Methods). Bar plot in the bottom panel shows overall changes of dust event frequency (grey) in three regions and the contributions from each controlling factor, i.e., precipitation (purple), bareness (green), and surface wind speed (orange). Error bars denote one standard deviation among 16 models. CMIP5 model simulated DOD variations are shown in cyan dots, with error bars denoting one standard deviation among the seven models used here. Maps were generated using the NCAR Command Language (NCL; https://www.ncl.ucar.edu/), version 6.2.1.

CMIP5 models simulate changes of the climate system in response to different greenhouse gas emission scenarios. We select models for which precipitation, bareness (or LAI), and surface wind speed interactively respond to climate changes (i.e., radiation, temperature, soil moisture, etc.). Using the regression model and projected changes of precipitation, bareness, and surface wind speed from 16 CMIP5 models (Supplementary Table S1), changes of dust event frequency due to climate change during 2051–2100 under the RCP 8.5 scenario with reference to that in the historical level (1861–2005) are examined (Fig. 5).

The dust event frequency reduces in the northern Great Plains and eastern U.S. in all seasons, with the largest reduction in spring, largely produced by the increase of precipitation and decrease of bareness (Fig. 5 and Supplementary Fig. S8). Dust event frequency also decreases in the western U.S. in winter and spring, but increases in the southwestern U.S in summer and fall. An increase of frequency up to 5% (about five more dusty days) is located in southern Texas in spring and extends to the central Plains in summer and persists to fall. Such an increase is largely associated with precipitation reduction and an increase of bareness due to vegetation decay, while an increase of surface wind in spring and summer also plays an important role (Fig. 5 and Supplementary Fig. S8).

The differences among CMIP5 models in changes in precipitation, bareness, and surface wind in the late twenty-first century lead to the spread of dust event frequency variations projected by the regression model. While most models agree with the increase of precipitation in the northern Great Plains from winter to spring and thus a reduction of dust event frequency, the variations of dust event frequency associated with summer precipitation change in the southern Great Plains have less consistency among the models (Fig. 5 and Supplementary Fig. S8). Models also have large disagreement on the direction of how bareness (or LAI) will change, especially over the southern Great Plains, and such a disagreement extends to the northern Great Plains in summer and fall. On the other hand, the increase of dust event frequency produced by an increase of surface wind over the southern Great Plains in spring and summer are consistently projected by the models, in association with a projected intensification of the Great Plains low-level jet^37, 38^. Despite the large spread of the regression, there is broad agreement in the increase of dust event frequency in the southern Great Plains in summer and a decrease in the northern Great Plains in spring.

We show that future dust activity is influenced by climate change-induced variations of precipitation and surface wind speed. The effects of carbon fertilization due to increasing CO_2_ concentrations compete with the influence of climate change (e.g., precipitation, radiation, and temperature) on vegetation growth. An increase of surface bareness in dry years may further amplify the influences of drought and increase dust activity. Together, these factors constrain the variations of dust activity.

What is the projection of the CMIP5 models on the variation of dust activity? Most models do not save DOD as an output, so we used monthly dust load and converted it to DOD (see Methods). Among the 16 models, only seven models used an interactive dust scheme and have output of dust load (Supplementary Table S1). Among these models, three simulated the magnitude of DOD in the U.S. that is comparable to the Aqua DOD from spring to fall (differences are within one order of magnitude), while other models underestimate DOD in all seasons (Supplementary Fig. S9). The spatial pattern of DOD is also not well captured by the models. Summer is the season when the ensemble mean shows better agreement with observations in terms of both magnitude and spatial pattern.

CMIP5 models project an overall dustier future than that projected from the regression analysis (Fig. 5 and Supplementary Fig. S10). DOD increases in most of the U.S. in spring, with a center over the southern Great Plains (Supplementary Fig. S10). The dustier region extends further north in summer, although some models project a decrease of DOD over the northern Great Plains and the northwest coast, while in fall the increase of DOD is largely over the southern U.S. with a weak decrease in the north.

## Discussion

This is an early attempt to project future changes in dust activity in the U.S. caused by increasing greenhouse gases, and the projections are based on the understanding of the controlling factors affecting dust event frequency in the present-day climate. Changes in land use associated with anthropogenic activities are not included in our regression model although some information is implicitly included in the simulation of vegetation coverage by some CMIP5 models (about 14 models, Supplementary Table S1) for future projections. While studies found changes of land use played an important role in the dust emission in the western U.S. in the past 200 years^39^, previous modeling studies have suggested that the influences of land use on future dust emission are minor compared to climate change^6^. The projected global land use change from now to the end of the twenty-first century is also much smaller than that from preindustrial levels to the present day^40^. However, whether dramatic land use change such as that before and during the Dust Bowl will occur over the Great Plains is hard to predict, depending on climate variability, irrigation technology and farming method, and economic conditions^36^. The non-linear interactions among precipitation, bareness, and surface wind variations are also not included. The uncertainties associated with CMIP5 projections of the three controlling factors also limit the accuracy of our projections, especially when the factor is identified as the dominant contributor to the variations of dust event frequency, e.g., LAI and precipitation in the Great Plains (Figs 3 and 5, Supplementary Fig. S8).

Finally, the projected increase of dust event frequency in the southern Great Plains during the warm season in the late twenty-first century may feed back to and potentially amplify the projected dry condition there as during previous mega-droughts^41–43^.

## Materials and Methods

### Data

Daily and monthly dust optical depth (DOD) derived from MODIS Deep Blue (M-DB2) aerosol products from Aqua satellite (level 2, collection 6) from 2003–2015 are used. The method to calculate DOD is discussed by refs 44–46. The horizontal resolution of DOD data is 0.1° by 0.1° grid. The quality of MODIS Deep Blue aerosol optical depth (AOD) and DOD are evaluated against AErosol RObotic NETwork^47^ (AERONET) stations over North America. Overall, MODIS slightly underestimates AOD and DOD (see discussion in Supplementary information and Supplementary Fig. S1).

Monthly precipitation data from the Precipitation Reconstruction over Land^48^ (hereafter PRECL) on a 1° by 1° resolution is used to calculate seasonal means from 2003–2015. Daily precipitation from Version 7 of Tropical Rainfall Measuring Mission (TRMM) Multi-satellite Precipitation Analysis (TMPA) daily product^49^ (3B42) is used for two case studies on drought years. TRMM covers latitudes from 50°S to 50°N with a spatial resolution of 0.25° by 0.25° and is available from 1998 to present.

Surface bareness is calculated from leaf area index (LAI) using the following equation,

1 $$Bareness=exp(-1\times LAI).$$

Monthly LAI data from Advanced Very High Resolution Radiometer^50, 51^ (AVHRR) on a 0.05° by 0.05° horizontal resolution is used to calculate seasonal mean LAI and then converted to seasonal mean bareness. AVHRR LAI is available from 1981 to present.

The monthly mean of 10 m surface wind from the North American Regional Reanalysis^52^ (NARR) is used. NARR provides high spatial (~32 km horizontally) resolution variables over North America from 1979 to present.

### CMIP5 model output

Precipitation, LAI, and surface wind speed from 16 CMIP5 models (Supplementary Table S1) are used as input for the regression model to calculate the variations of dust event frequency. Monthly output from both the historical run (1861–2005) and the RCP 8.5 run (2051–2100) are used to generate seasonal means. Seven out of the 16 models calculated dust concentration interactively (i.e., not prescribed) in the model and are used to derive DOD shown in Fig. 5 and Supplementary Figs S9,10 (see details below).

### Dust event frequency and regional indices

The dust event frequency in each season is calculated from Aqua daily DOD data. The total number of days with DOD anomaly (with reference to climatological mean) greater than one standard deviation is divided by the total days of the season to get the frequency for each season.

Since the dust emission and transport is coupled with both land surface features such as bareness and also meteorological conditions such as surface winds and precipitation, its seasonal cycle varies over regions. To understand the dust activities we selected three regions, e.g., the southwestern U.S. (WST1; Box1 in Fig. 1), the southern Great Plains (SGP; Box 3) and the northern Great Plains (NGP; Box 4) to calculate dust event frequency in each region (Fig. 2) and regional averaged frequency (Fig. 5, Supplementary Fig. S5). These regions have quite different seasonality of the distribution of dust event frequency (Supplementary Fig. S3) and seasonal cycle of DOD (Supplementary Fig. S4). The calculation of multiple-linear regression introduced more missing values in the southwestern U.S., so a larger domain, the western U.S. (WST; Boxes 1 and 2), is used later when examining the results from the regression analysis.

### Multiple linear regression model

The multiple-linear regression coefficients are calculated by regressing the dust event frequency onto standardized seasonal mean precipitation from PRECL, bareness derived from AVHRR LAI, and NARR surface wind speed from 2004 to 2015 for each season. All the data are interpolated to a 1° by 1° grid before the calculation. Over regions where values were missing for any of the explanatory variables (i.e., precipitation, bareness, and surface wind speed) or dust event frequency, the regression coefficients are set to missing values. The missing value in the southwestern U.S. is mainly related to missing data from LAI (Fig. 3). Since these controlling factors are not completely independent of each other, i.e., precipitation can influence vegetation growth by affecting soil moisture and radiation and thus bareness, and surface wind can interact with precipitation, the collinearity among these factors are examined by calculating variable inflation factor (VIF) (e.g., ref. 53), and in most regions the VIF is below 3 (not shown), suggesting a low collinearity (5–10 is usually considered high).

Besides comparing the calculated dust event frequency with observations in Fig. S5, we also examined the capability of the model by comparing the calculated dust event frequency from 1995–2014 with the coarse mode aerosol optical depth (COD) at 500 nm from an AERONET site at Sevilleta, New Mexico (Fig. S6). The COD is processed by the Spectral Deconvolution Algorithm^54^. Sevilleta site (34.4°N, 106.9°W) is the only available site with more than 10 years of records with proximity to major dust sources in the U.S. The variations of dust event frequency from some nearby grids show significant positive correlations with the COD data from spring to fall (Supplementary Fig. S6).

The projection of dust event frequency change used the regression coefficients derived from 2004–2015 and CMIP5 model output of precipitation, LAI, and surface wind speed. All model output are interpolated to a 2° by 2.5° grid. The differences between the historical run (1861–2005 average) and that of the RCP 8.5 run for the late half of the twenty-first century (2051–2100) are standardized by the standard deviation of the historical run for each explanatory variable. The projected change reveals how the frequency of dust event will vary with reference to the historical conditions (mean and standard deviation).

The agreement between the models is calculated by comparing the sign of the variable (e.g., the changes of dust event frequency) in an individual model with that of the multi-model ensemble mean. An agreement of 100% denotes that the direction of change (i.e., RCP8.5 run minus the historical run) projected by all individual models are the same as the ensemble mean, and a 62.5% agreement denotes 10 models show the change with the same sign as the multi-model mean.

### Bootstrap test

Bootstrap resampling is used to test the statistical significance of the regression coefficients. First the regression error or residual (**ε**) is calculated by subtracting the calculated dust event frequency from the observed dust event frequency,

2 $${{\rm{\varepsilon }}}_{{\rm{i}}}={{{\rm{y}}}^{{\rm{obs}}}}_{{\rm{i}}}-{{{\rm{y}}}^{{\rm{reg}}}}_{{\rm{i}}},$$

where i = 1, 2, 3, …, 12, denotes 12 years, and **y** ^**obs**^_**i**_ denotes the observed dust event frequency (two-dimensional), **y** ^**reg**^_**i**_ denotes calculated dust event frequency (two-dimensional) using observed precipitation, bareness, and surface wind speed and the regression coefficients. Then the errors are randomly selected (**ε** ^**radom**^_**j**_, where j = 1, 2, 3, …, 12) and added back to the calculated dust event frequency to get a slightly different dust event frequency series $({{\rm{y}}}_{{\rm{i}}}^{\ast })$,

3 $${{\rm{y}}}_{{\rm{i}}}^{\ast }={{{\rm{y}}}^{{\rm{reg}}}}_{{\rm{i}}}+{{{\rm{\varepsilon }}}^{{\rm{radom}}}}_{{\rm{j}}},$$

where i = 1, 2, 3, …, 12. New regression coefficients are then calculated using the same observations. The above process is repeated 10,000 times to get 10,000 groups of coefficients. The 90% confidence intervals are determined using bias-corrected accelerated percentile intervals^55^. The coefficients are considered significant at the 90% confidence level if the confidence intervals do not include zero. Other methods such as two-tailed student t-test, and permutation of explanatory variables are also examined, and results are similar to that from the bootstrap test.

### Conversion of modeled dust load to DOD

Using the relationship derived in ref. 56 (their equation 1), we converted the output of dust load from the seven CMIP5 models (Supplementary Table S1) to DOD as the following,

4 $$\tau =M\times e,$$

where *τ* is DOD at 500 nm, *M* is the load of dust in unit of (g m^−2^), and *e* = 0.6 m^2^ g^−1^ is the mass extinction efficiency. We compared the derived DOD with the DOD output from the GFLD-CM3 model^57^ for one historical simulation, and the overall pattern is well captured by this method, with pattern correlations (for the 1861–2005 average) in a range from 0.94 in winter to 1.00 in summer and fall. The magnitude of DOD is underestimated by 26–36% over the southern Great Plains, 16–33% over the northern Great Plains and 5–27% over the western U.S.

### Data availability

The MODIS Deep Blue aerosol products were acquired from the Level-1 and Atmosphere Archive and Distribution System (LAADS) Distributed Active Archive Center (DAAC), located in the Goddard Space Flight Center in Greenbelt, Maryland (https://ladsweb.nascom.nasa.gov/). PRECL precipitation data are download from: http://www.esrl.noaa.gov/psd/, while TRMM precipitation data are available from: http://disc.sci.gsfc.nasa.gov/gesNews/trmm_v7_multisat_precip. AVHRR leaf area index data are available at: ftp://eclipse.ncdc.noaa.gov/pub/cdr/lai-fapar/files/.

NARR data are downloaded from: https://www.esrl.noaa.gov/psd/data/gridded/data.narr.html. CMIP5 data are downloaded from: https://pcmdi.llnl.gov/projects/esgf-llnl/. AERONET data are downloaded from: https://aeronet.gsfc.nasa.gov/.

## Electronic supplementary material

Supplementary Information
