# Supplementary material for: Projection of American dustiness in the late 21st century due to climate change
Source: Sci Rep. 2017 Jul 17;7:5553. doi: 10.1038/s41598-017-05431-9 (PMC5514090; doi:10.1038/s41598-017-05431-9)
Supplement: Supplementary file 1 — Supplementary Information [file 41598_2017_5431_MOESM1_ESM.pdf]

Projection of American dustiness in the late 21st century due to climate change

Bing Pu[1,2] and Paul Ginoux[2]

[1]Atmospheric and Oceanic Sciences Program, Princeton University,

Princeton, New Jersey 08544 USA

[2]NOAA Geophysical Fluid Dynamics Laboratory, Princeton, New Jersey 08540 USA

Correspondence to: Bing Pu, bpu@princeton.edu

Supplementary information

Table S1 CMIP5 models used in this study. Models with interactive dust emission scheme are marked with stars (*). Models tagged with plus signs (+) considered anthropogenic land use/land cover change in their vegetation prediction.

Model	lat/lon resolution	Dust emission scheme	Dynamic Vegetation	Model reference
BCC-CSM1.1	1.1°×1.1°	offline	Y	Wu et al. (2013) [1]
BNU-ESM	2.8°×2.8°	offline	Y	Ji et al. (2014) [2]
CanESM2*	2.8°×2.8°	Reader et al. (1999)[3]; Croft et al. (2005) [4]	N^+	Arora et al. (2011) [5]
GFDL-CM3*	2.0°×2.5°	Ginoux et al. (2001)[6]	Y^+	Donner et al. (2011) [7]
GFDL-ESM2G	2.0°×2.5°	offline	Y^+	Dunne et al. (2013) [8]
GFDL-ESM2M	2.0°×2.5°	offline	Y^+	Dunne et al. (2013)[8]
HadGEM2-CC*	1.2°×1.8°	Marticorena and Bergametti (1995)[9]	Y^+	Collins et al. (2011) [10]
HadGEM2-ES*	1.2°×1.8°	Marticorena and Bergametti (1995)[9]	Y^+	Collins et al. (2011)[10]
IPSL-CM5A-LR	1.9°×3.75°	offline	N^+	Dufresne et al. (2013) [11]
IPSL-CM5A-MR	1.25°×2.5°	offline	N^+	Dufresne et al. (2013) [11]
IPSL-CM5B-LR	1.9°×3.75°	offline	N^+	Dufresne et al. (2013)[11]
MIROC-ESM*	2.8°×2.8°	Takemura et al. (2000)[12]	Y^+	Watanabe et al. (2011)[13]
MIROC-ESM-CHEM*	2.8°×2.8°	Takemura et al. (2000)[12]	Y^+	Watanabe et al. (2011)[13]
MPI-ESM-LR	1.9°×1.9°	offline	Y^+	Giorgetta et al. (2013) [14]
MPI-ESM-MR	1.9°×1.9°	offline	Y^+	Giorgetta et al. (2013) [14]
NorESM1-M*	1.9°×2.5°	Seland et al. (2008)[15]	N^+	Bentsen et al. (2013) [16]

1. Validation of MODIS DOD against observation

Figure S1 shows the comparisons between MODIS Deep Blue aerosol optical depth (AOD) and AErosol RObotic NETwork (AERONET) AOD (top and middle), and between MODIS DOD and AERONET coarse mode aerosol optical depth (COD; bottom). AERONET COD is processed by the Spectral Deconvolution Algorithm[17]. We used an evaluation method following ref 18 (their Fig. 11) for AOD and COD errors. The AERONET Level 2 (quality assured) 10 minutes AOD and COD (500 nm) are extracted for Aqua equatorial crossing time (1:30 PM) plus or minus 30 minutes, and are considered if there is at least 2 measurements. We select AERONET sites over North America within a spatial radius of 20 km of MODIS measurement. 87 AERONET sites are used. Total number of valid data is about 12850. In box-whisker plots (Fig. S1 middle and bottom), all collocated MODIS and AERONET data are grouped into bins of 500 measurements. The last bin will contain a larger number of values corresponding to the remaining of the division.

MODIS slightly underestimated AOD for most of the AOD ranges (Fig. S1 top and middle) but slightly overestimates in some regions such as over southern California (Fig. S1 top). DOD is slightly overestimated for values less than 0.02, but underestimated for values above 0.02 (Fig. S1 bottom). We masked DOD data with values less than 0.02 for quality purpose and also to distinguish dust events from background aerosols. Our results are not sensitive to such a screening.

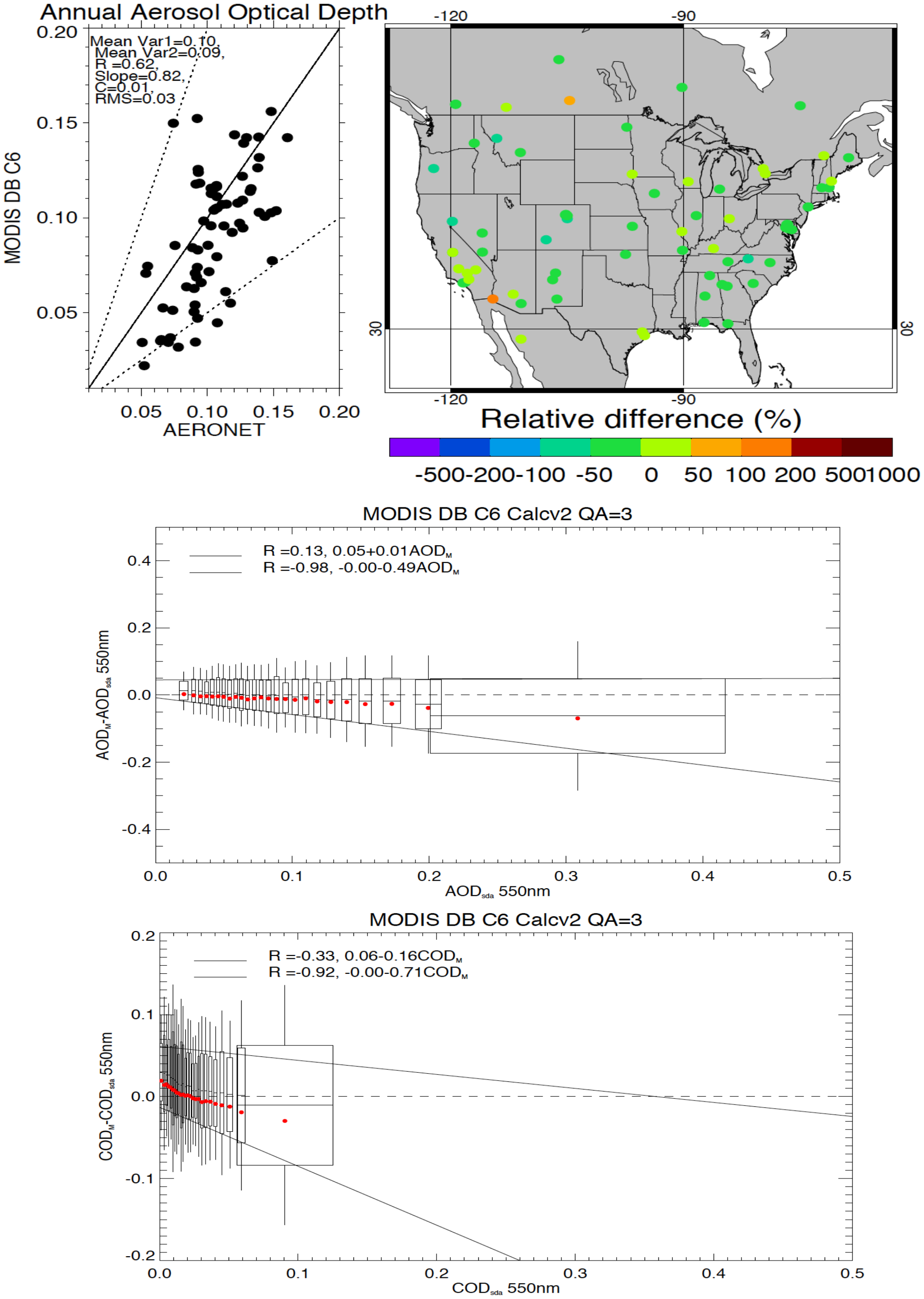

Figure S1. Comparison between MODIS AOD and AERONET AOD for each AERONET station over North America (top), grouped AOD error (MODIS-AERONET versus AERONET, middle), and grouped coarse mode aerosol optical depth (COD) error (bottom). For each box-whisker, its width is 1σ of the AOD (COD) bin, while its height, whiskers, middle line and red dots are the 1σ, 2σ, mean, and median of AOD (COD) error, respectively. The envelope of estimated errors are solid and the one-one line (zero error) is dashed. Maps were generated using the Interactive Data Language (IDL; http://harrisgeospatial.co.uk/ProductsServices/IDL.aspx), version 8.0.1.

2. Supplementary figures for the analysis on dust activity and future projection

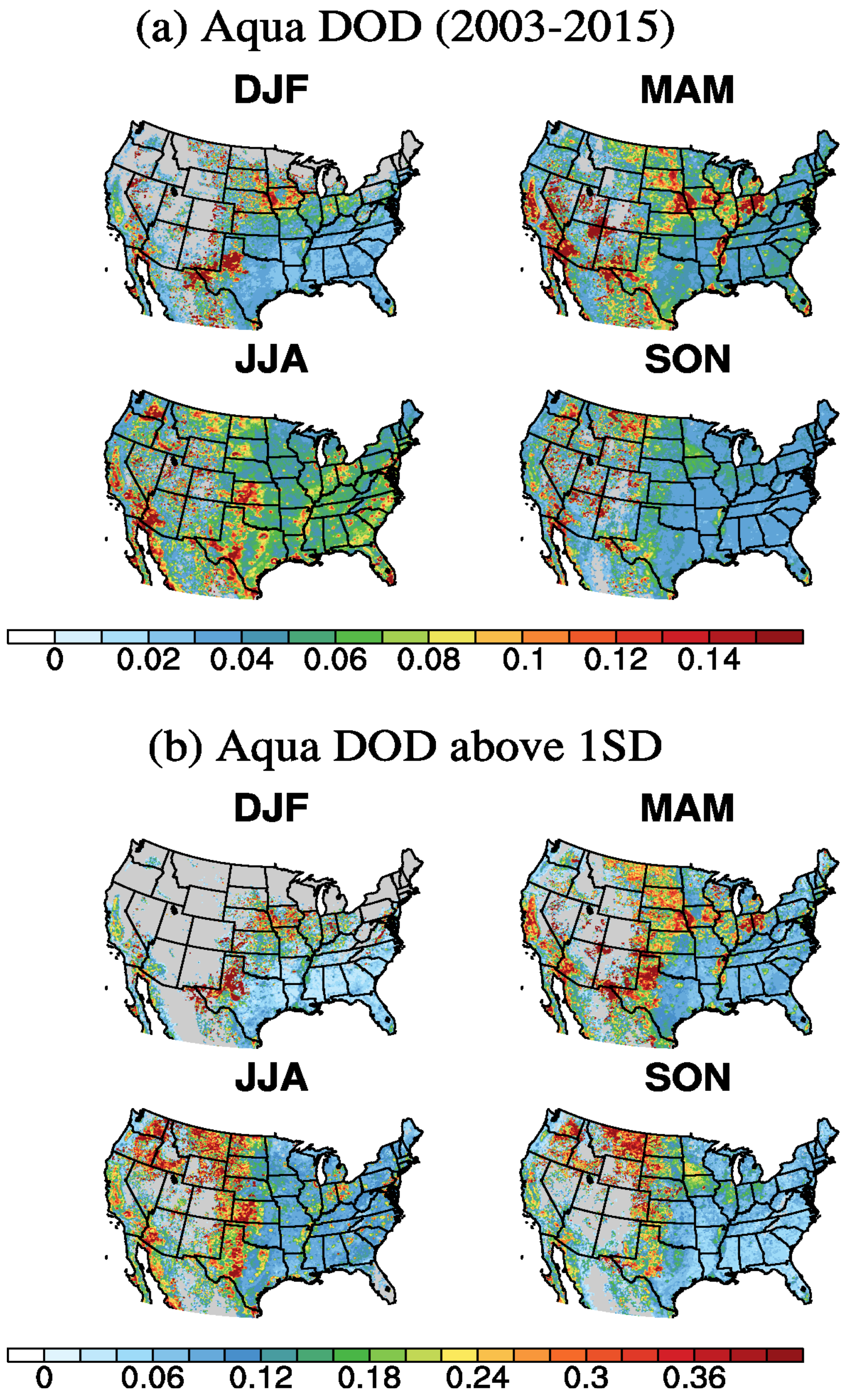

Figure S2. Climatology of dust optical depth (DOD) from Aqua. DOD averaged from 2003 to 2015 in each season (upper panel) and averages of daily DOD above 1 standard deviation (bottom panel). Missing values are plotted in grey. Maps were generated using the NCAR Command Language (NCL; https://www.ncl.ucar.edu/), version 6.2.1.

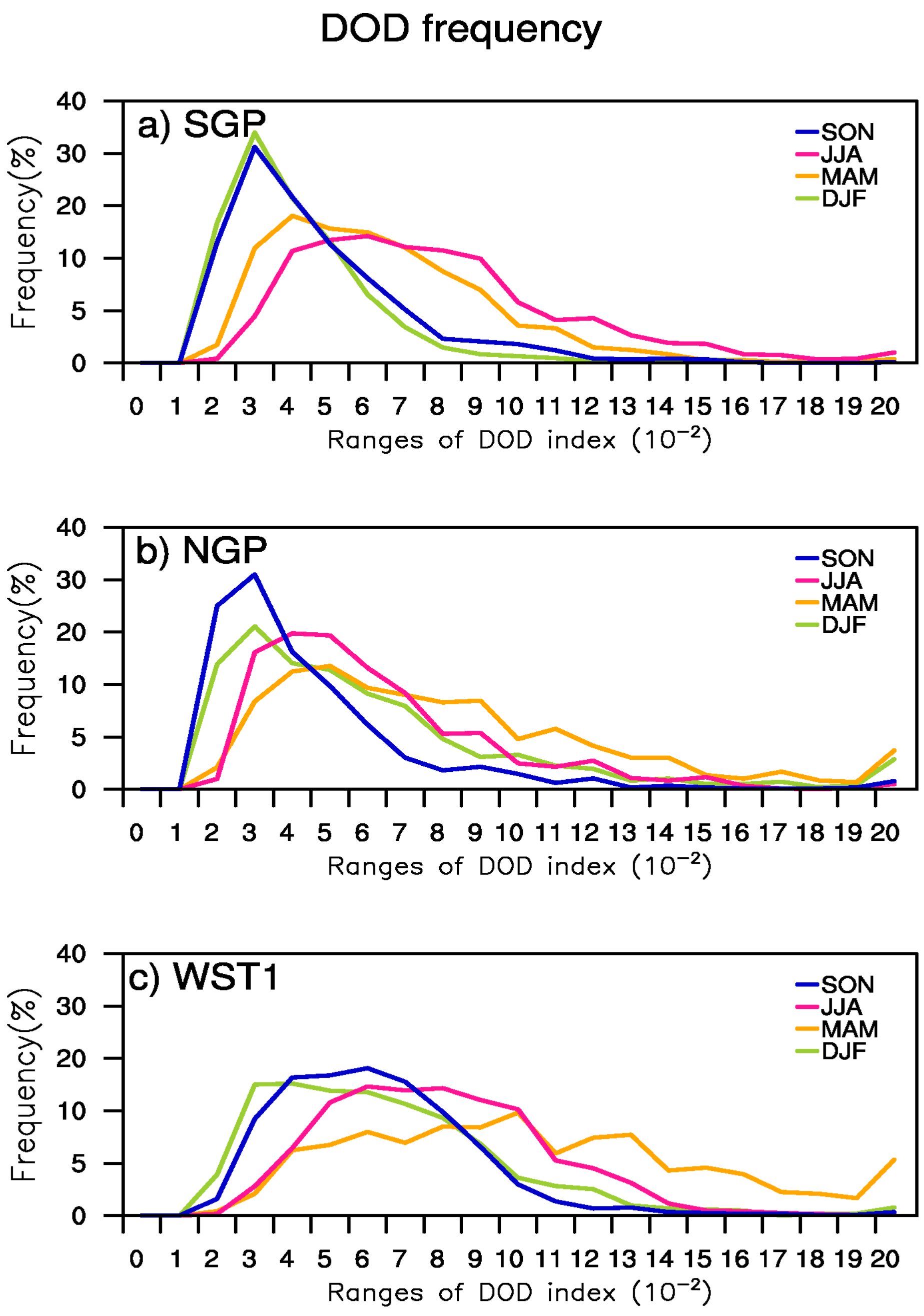

Figure S3. Frequency distribution of regional averaged daily DOD. (a) The southern Great Plains (Box 3 in Fig. 1), (b) northern Great Plains (Box 4 in Fig. 1), and (c) southwestern U.S. (Box 1 in Fig. 1).

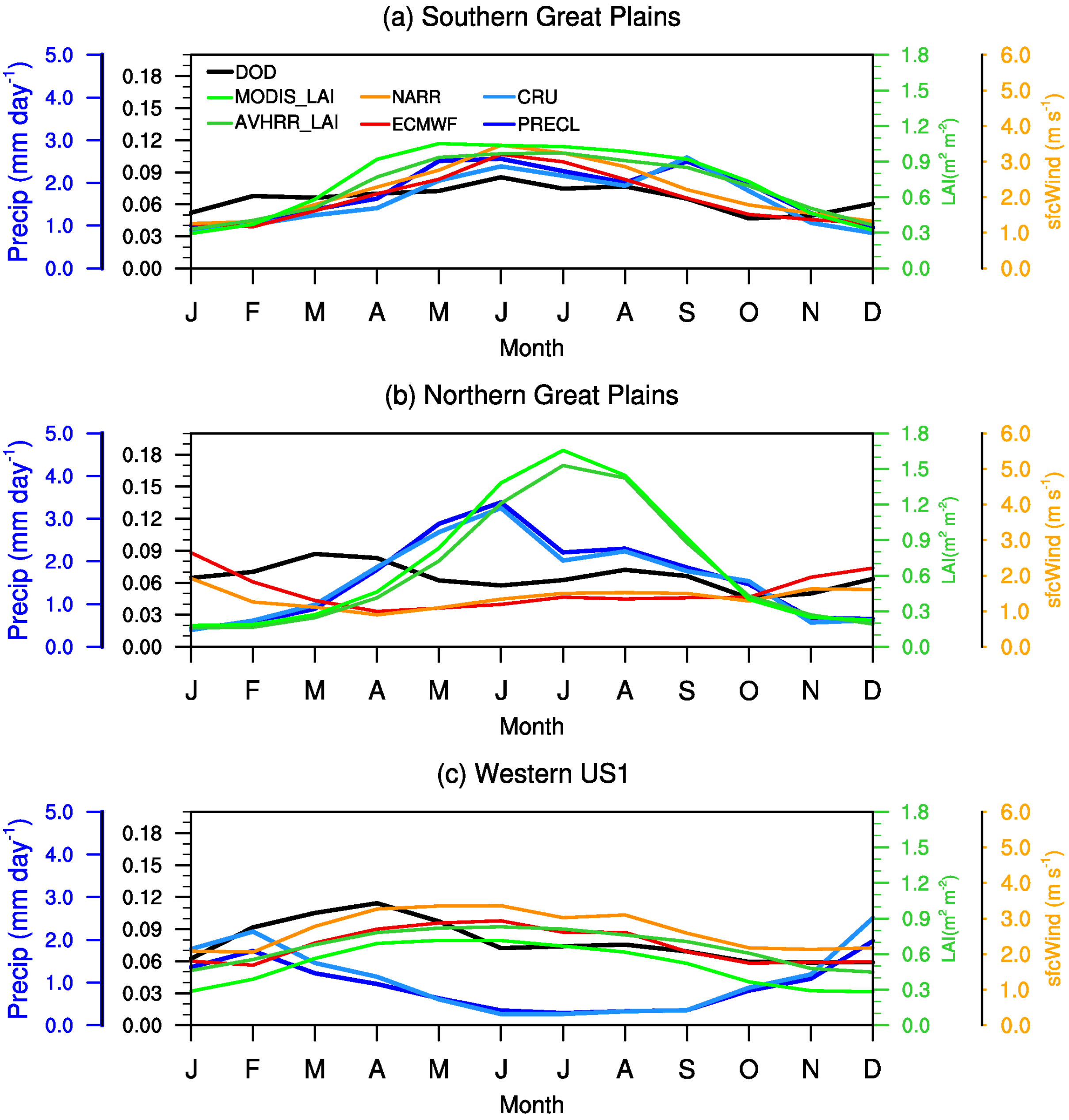

Figure S4. Seasonal variations of DOD and environmental variables. Seasonal cycle of MOIDS Aqua DOD (black), LAI (light and dark green), precipitation (light and dark blue) and surface wind (red and orange) from different datasets averaged over (a) the southern Great Plains, (b) northern Great Plains, and (c) southwestern U.S. (Box 1).

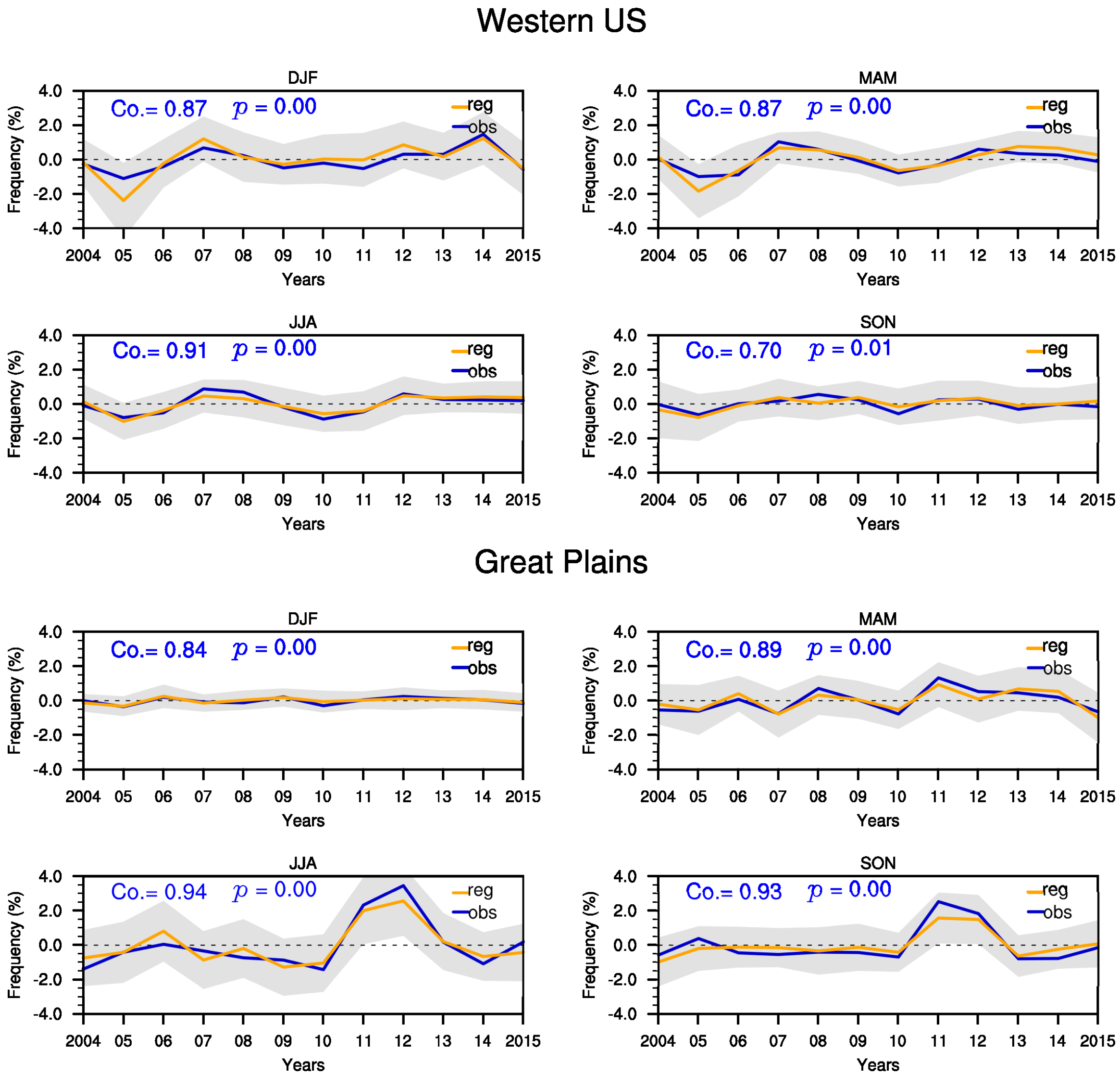

Figure S5. Calculated and observed dust event frequency from 2004 to 2015. Dust event frequency from observation (blue) and calculated from the regression model (regression) averaged for the Western U.S. (Boxes 1 and 2 in Fig. 1) and the Great Plains (Box 3 and Box 4). Time series are shown in anomalies (with reference to the 2004-2015 mean). The correlations between the observed and calculated time series are shown at the top left corner of each plot, and the shading denotes the 90% confidence interval (t-test) of the regression.

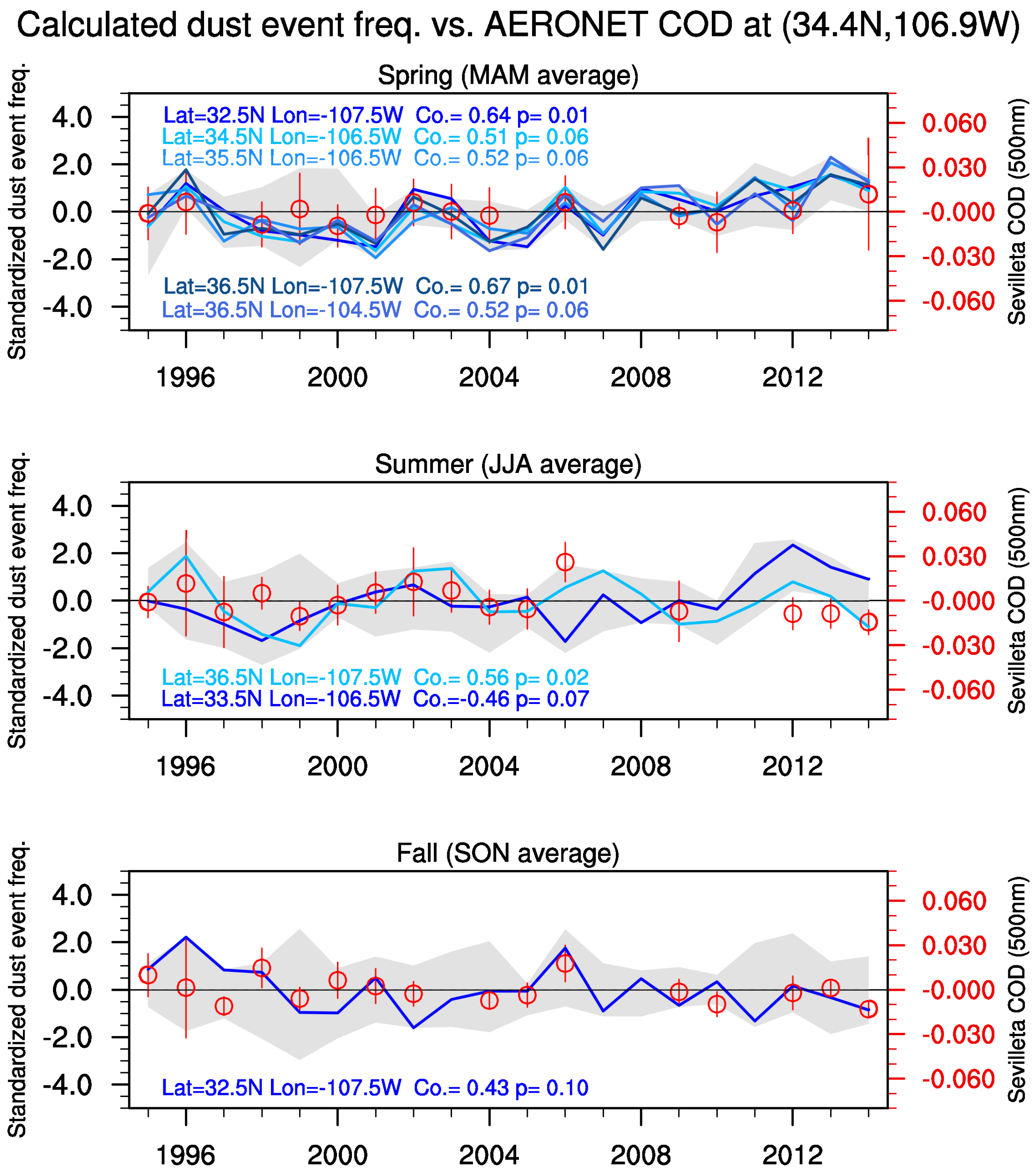

Figure S6. Comparison between dust event frequency calculated from the regression model and observed coarse mode aerosol optical depth (COD) at 500 nm from AERONET site at Sevilleta. COD is plotted in red cycles with error bar indicating ±one standard deviations. Dust event frequency is calculated using the same method as in Fig. S5 but for 1995-2014. The grey shading denotes the range of the dust event frequency among the 25 grids near the Sevilleta site (between 32.5-36.5N and 104.5-108.5W), and the blue lines are from the grids where the correlation with Sevilleta COD is significant at or above the 90% confidence level. All time series of dust event frequency are standardized.

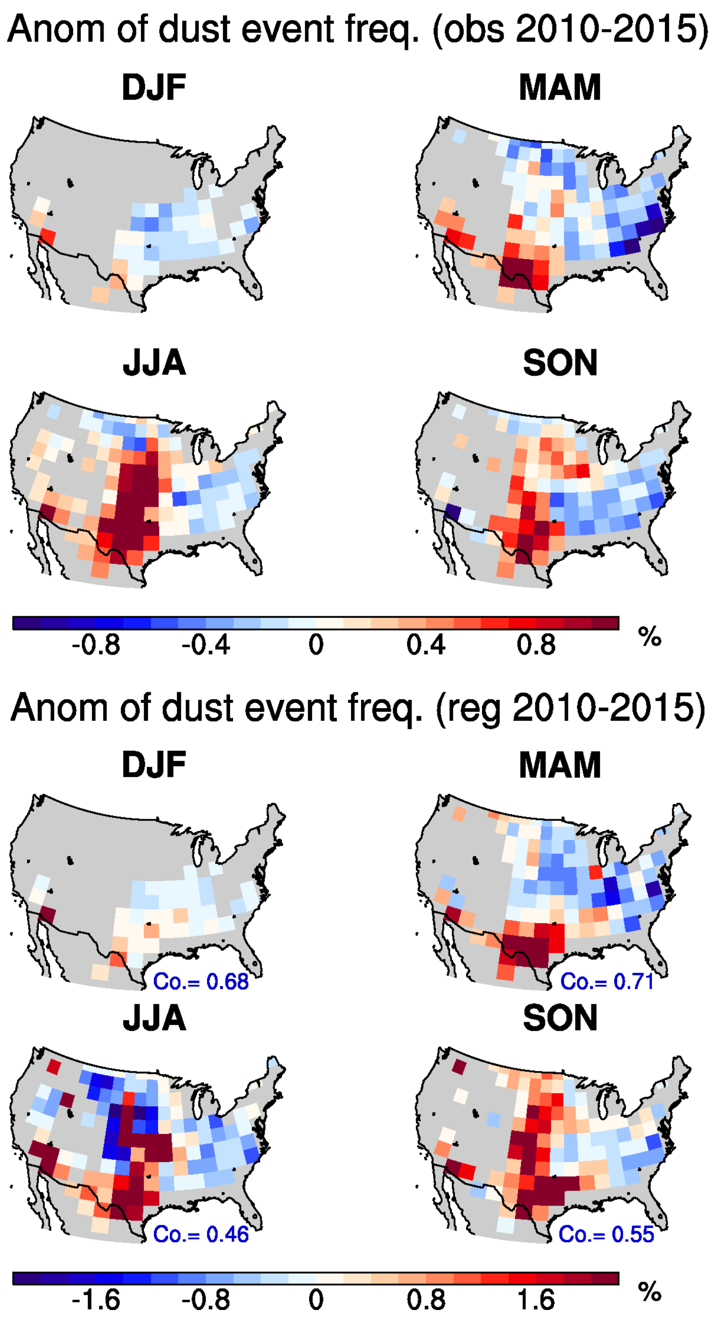

Figure S7. Anomalies of dust event frequency from the observation (upper panel) and from the regression model using CMIP5 output (bottom panel). The observed anomalies are obtained by removing the mean of dust event frequency during 2004-2015. The regression is calculated using the regression coefficients shown in Fig. 3 and modeled changes of precipitation, bareness, and surface wind speed between CMIP5 historical run (1861-2005) and RCP8.5 run (2010-2015). The pattern correlation (centered) coefficients between observed dust event frequency and that from the regression model are shown at the bottom corner of the each plot in the lower panel. Maps were generated using the NCAR Command Language (NCL; https://www.ncl.ucar.edu/), version 6.2.1.

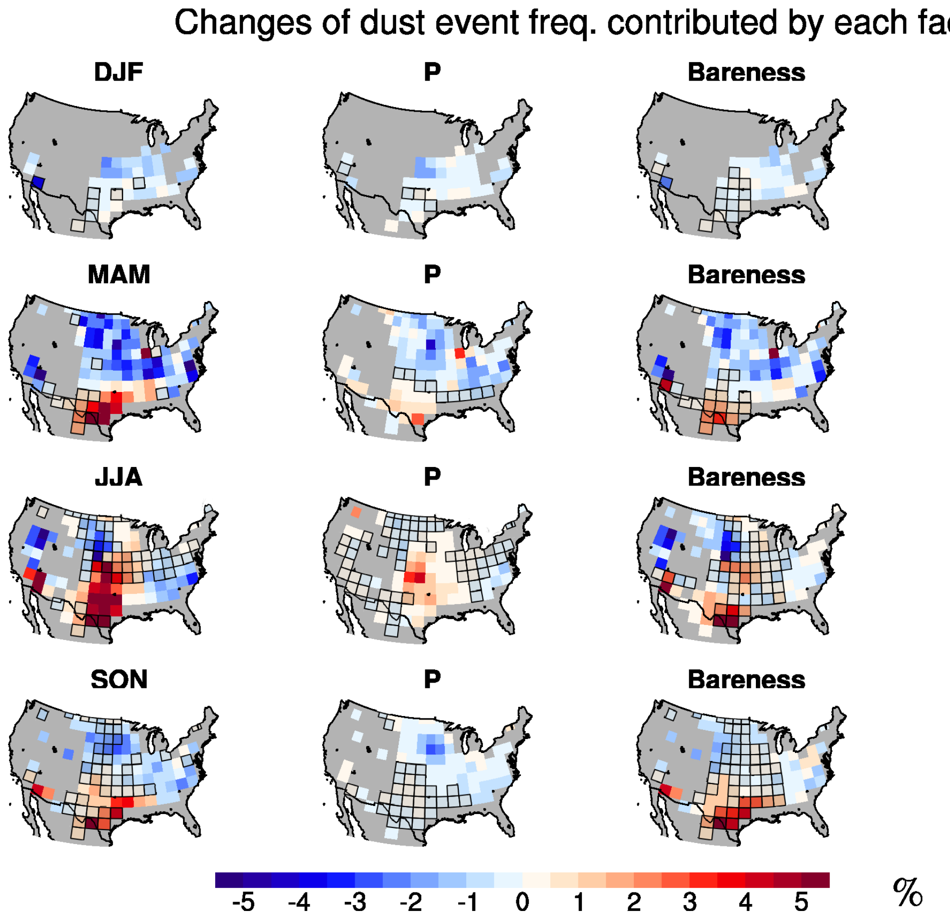

Figure S8. Changes of the dust event frequency under the RCP 8.5 scenario (2051-2100) with reference to the historical run (1861-2005). Results are calculated using the regression model and output from 16 CMIP5 models (See methods). The left column shows the changes of dust event frequency (same as Fig. 4), while the other columns show changes of frequency associated with changes of precipitation (second column), bareness (third) and surface wind speed (fourth). Shaded boxes denote area where the agreement among the models is lower than 62.5% (i.e., when less than 10 model shows the anomaly with the same sign as the multi-model mean). Maps were generated using the NCAR Command Language (NCL; https://www.ncl.ucar.edu/), version 6.2.1.

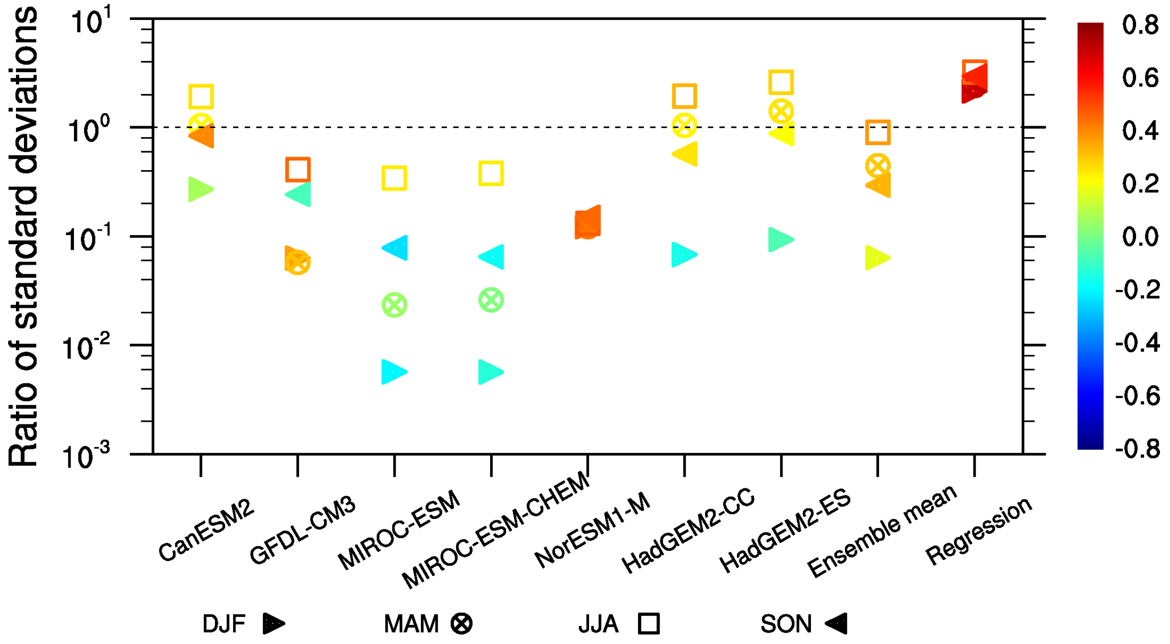

Figure S9. Pattern statistics comparing DOD from CMIP5 models with that from observation in the U.S. and part of Mexico (from 25° to 49°N, 66.25° to 126.25°W). Label on the X-axis shows individual models and multi-model mean. Y-axis shows the ratio of pattern standard deviations between model climatology (2004-2015) and that of MODIS Aqua, which reveals the relative amplitude of the simulated DOD versus observation. The color denotes pattern correlation (centered) between each model and Aqua DOD for the domain mentioned above. The results from the regression model are also included, i.e., comparing the patterns of observed and calculated anomalies of dust event frequency averaged from 2010-2015 (Fig. S7).

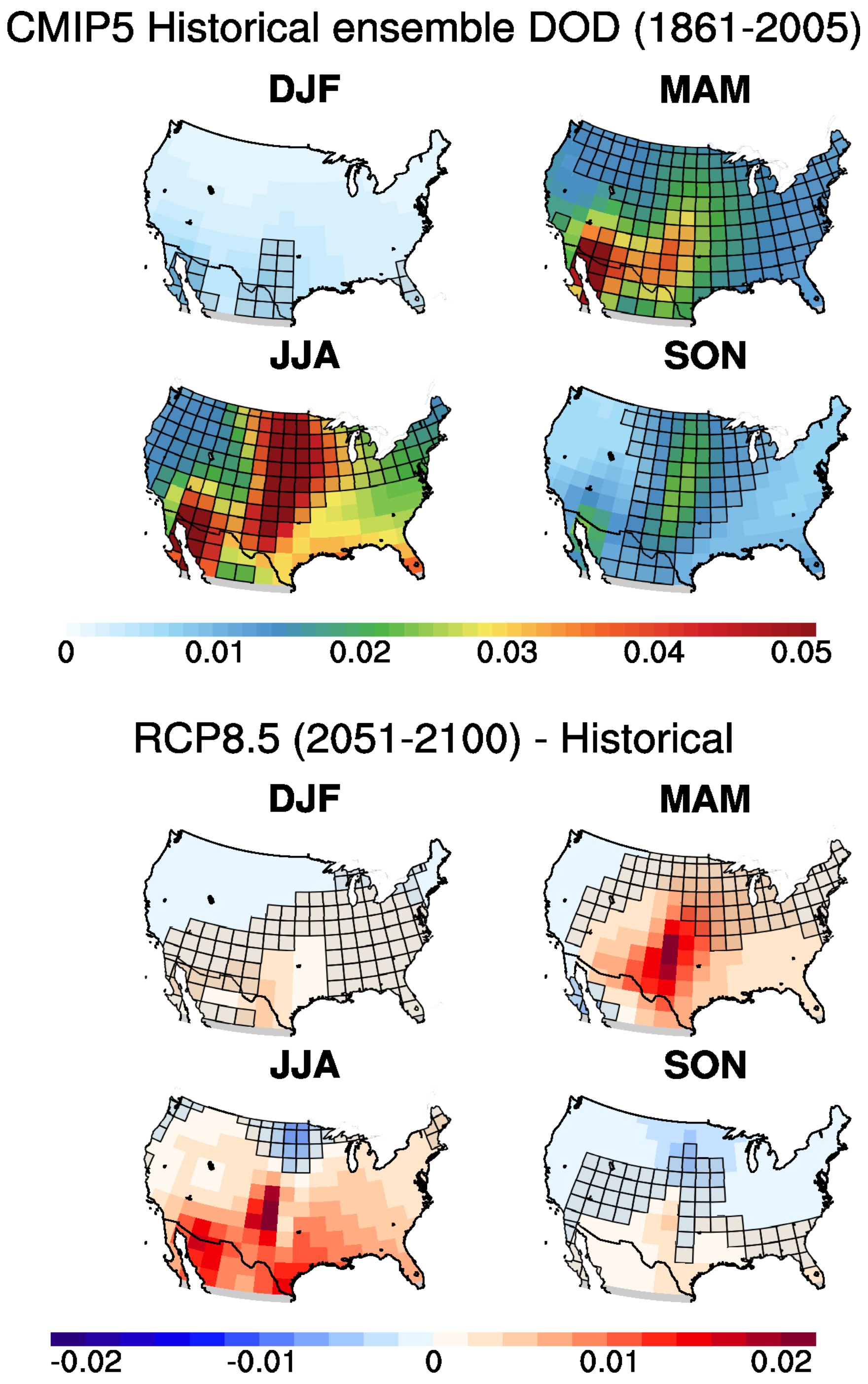

Figure S10. DOD from CMIP5 multi-model mean. Model ensemble mean for each season averaged from historical run (1861-2005 average; upper panel) and the differences between RCP8.5 simulation (2051-2100 average) and historical run (bottom panel). Shading in the upper panel denotes area where the multi-model ensemble mean is less than one standard deviation among all the seven models, while in the bottom panel it denotes where model agreement is less than 62.5%. Maps were generated using the NCAR Command Language (NCL; https://www.ncl.ucar.edu/), version 6.2.1.

References

1 Wu, T. W. *et al.* Global carbon budgets simulated by the Beijing Climate Center Climate System Model for the last century. *J Geophys Res-Atmos* **118**, 4326-4347, doi:10.1002/jgrd.50320 (2013).

2 Ji, D. *et al.* Description and basic evaluation of Beijing Normal University Earth System Model (BNU-ESM) version 1. *Geosci Model Dev* **7**, 2039-2064, doi:10.5194/gmd-7-2039-2014 (2014).

3 Reader, M. C., Fung, I. & McFarlane, N. The mineral dust aerosol cycle during the Last Glacial Maximum. *J Geophys Res-Atmos* **104**, 9381-9398, doi:Doi 10.1029/1999jd900033 (1999).

4 Croft, B., Lohmann, U. & von Salzen, K. Black carbon ageing in the Canadian Centre for Climate modelling and analysis atmospheric general circulation model. *Atmos Chem Phys* **5**, 1931-1949 (2005).

5 Arora, V. K. *et al.* Carbon emission limits required to satisfy future representative concentration pathways of greenhouse gases. *Geophys Res Lett* **38**, doi:10.1029/2010gl046270 (2011).

6 Ginoux, P. *et al.* Sources and distributions of dust aerosols simulated with the GOCART model. *J Geophys Res-Atmos* **106**, 20255-20273, doi:Doi 10.1029/2000jd000053 (2001).

7 Donner, L. J. *et al.* The Dynamical Core, Physical Parameterizations, and Basic Simulation Characteristics of the Atmospheric Component AM3 of the GFDL Global Coupled Model CM3. *J Climate* **24**, 3484-3519, doi:10.1175/2011jcli3955.1 (2011).

8 Dunne, J. P. *et al.* GFDL's ESM2 Global Coupled Climate-Carbon Earth System Models. Part II: Carbon System Formulation and Baseline Simulation Characteristics. *J Climate* **26**, 2247-2267, doi:10.1175/Jcli-D-12-00150.1 (2013).

9 Marticorena, B. & Bergametti, G. Modeling the Atmospheric Dust Cycle .1. Design of a Soil-Derived Dust Emission Scheme. *J Geophys Res-Atmos* **100**, 16415-16430, doi:Doi 10.1029/95jd00690 (1995).

10 Collins, W. J. *et al.* Development and evaluation of an Earth-System model-HadGEM2. *Geosci Model Dev* **4**, 1051-1075, doi:10.5194/gmd-4-1051-2011 (2011).

11 Dufresne, J. L. *et al.* Climate change projections using the IPSL-CM5 Earth System Model: from CMIP3 to CMIP5. *Clim Dynam* **40**, 2123-2165, doi:10.1007/s00382-012-1636-1 (2013).

12 Takemura, T. *et al.* Global three-dimensional simulation of aerosol optical thickness distribution of various origins. *J Geophys Res-Atmos* **105**, 17853-17873, doi:Doi 10.1029/2000jd900265 (2000).

13 Watanabe, S. *et al.* MIROC-ESM 2010: model description and basic results of CMIP5-20c3m experiments. *Geosci Model Dev* **4**, 845-872, doi:10.5194/gmd-4-845-2011 (2011).

14 Giorgetta, M. A. *et al.* Climate and carbon cycle changes from 1850 to 2100 in MPI-ESM simulations for the Coupled Model Intercomparison Project phase 5. *J Adv Model Earth Sy* **5**, 572-597, doi:10.1002/jame.20038 (2013).

15 Seland, O., Iversen, T., Kirkevag, A. & Storelvmo, T. Aerosol-climate interactions in the CAM-Oslo atmospheric GCM and investigation of associated basic shortcomings. *Tellus A* **60**, 459-491, doi:10.1111/j.1600-0870.2008.00318.x (2008).

16 Bentsen, M. *et al.* The Norwegian Earth System Model, NorESM1-M - Part 1: Description and basic evaluation of the physical climate. *Geosci Model Dev* **6**, 687-720, doi:10.5194/gmd-6-687-2013 (2013).

17 O'Neill, N. T., Eck, T. F., Smirnov, A., Holben, B. N. & Thulasiraman, S. Spectral discrimination of coarse and fine mode optical depth. *J Geophys Res-Atmos* **108**, doi:10.1029/2002jd002975 (2003).

18 Levy, R. C. *et al.* The Collection 6 MODIS aerosol products over land and ocean. *Atmos Meas Tech* **6**, 2989-3034, doi:10.5194/amt-6-2989-2013 (2013).
